# How systematic reviews and meta-analyses based on individual participant data can inform trial design and conduct

**DOI:** 10.1186/1745-6215-14-S1-O94

**Published:** 2013-11-29

**Authors:** Jayne Tierney, Claire Vale, Jean-Pierre Pignon, Francois Gueffyier, Lisa Askie, Mike Clarke

**Affiliations:** 1 MRC Clinical Trials Unit Hub for Trials Methodology Research, London, UK; 2 Institut Gustave Roussy, Villejuif, France; 3 UMR5558, CNRS, Lyon, France; 4 NHMRC Clinical Trials Centre, Sydney, Australia; 5 All-Ireland Hub for Trials Methodology Research, Belfast, Ireland

## Introduction

Systematic reviews and meta-analyses provide an objective and reliable way of summarising trial results, and can inform both clinical practice and the design, conduct and reporting of trials. However, those based on aggregate data are sometimes limited by the availability of such data, while those based on more comprehensive individual participant data (IPD) can provide more detailed and reliable results. Also, IPD meta-analyses provide a resource for secondary hypothesis testing, which can produce further clinical insight, and rely on close collaboration with trials organisations worldwide. Thus, IPD meta-analyses have the potential to better inform new and on-going trials.

## Methods

Initially, we sought examples of IPD meta-analyses that have directly influenced the design and conduct of trials, via a workshop of international experts in the field, and subsequently, through this subgroup of workshop attendees. We also considered additional ways that IPD meta-analysis could impact on trials.

## Results

In terms of trial design, IPD meta-analyses results have informed the choice of comparators; definition of trial populations; sample size calculations and effect sizes to target. They have also been the catalyst for international collaboration on new trials; justified both continuing and stopping trial recruitment, and informed stratification of trial analyses. They have the potential to inform other aspects, such as the choice and definition of outcomes. We illustrate these impacts in a range of health care areas including cancer, cardiovascular disease, stroke and neonatal care.

## Conclusions

IPD meta-analyses have impacted on trials in various ways, but might be utilised more widely.

